# The effect of electroacupuncture on postoperative immunoinflammatory response in patients undergoing supratentorial craniotomy

**DOI:** 10.3892/etm.2013.1225

**Published:** 2013-07-16

**Authors:** GUOYAN LI, SHUQIN LI, BAOGUO WANG, LIXIN AN

**Affiliations:** 1 Department of Anesthesiology, Beijing Tiantan Hospital, Capital Medical University, Beijing 100050, Beijing, P.R. China; 2 Department of Anesthesiology, State Grid Beijing Electric Power Hospital, Beijing, P.R. China; 3 Department of Anesthesiology and Pain Management, Beijing Sanbo Brain Hospital, Capital Medical University, Beijing 100093, P.R. China

**Keywords:** electroacupuncture, supratentorial craniotomy, immune suppression

## Abstract

The aim of this study was to explore the effect of electroacupuncture (EA) on immune function in patients undergoing supratentorial craniotomy. We also examined whether point specificity in EA was present. The study involved 29 patients undergoing craniotomy. The patients were divided into three groups: a control (C, n=10), an EA (A, n=9) and a sham acupoints group (S, n=10). Blood samples were collected at the following time points: before anesthesia (T0), 4 h after the induction of anesthesia (T1), 1 day post-surgery (T2) and 2 days post-surgery (T3) to determine the levels of tumor necrosis factor-α (TNF-α), interleukin-8 (IL-8), interleukin-10 (IL-10), immunoglobulin M (IgM), IgA and IgG. Data were analyzed using SPSS 13.0 software. When comparing the levels of cytokines following surgery, we observed that the peripheral blood IL-8 levels in groups A and S were increased significantly compared with those of group C at 1 and 2 days after surgery. When comparing immunoglobulin levels after surgery, we established that the peripheral blood IgA levels in group C had decreased significantly compared to those of group A and group S 4 h after induction of anesthesia and 1 day after surgery. However, there was no significant difference between group A and group S. Compared with simple general anesthesia, acupuncture combined with anesthesia partially reduces immune suppression in the perioperative periods under the same conditions as the simple general anesthesia. Point specificity in EA was not present.

## Introduction

Major surgery induces a high risk of postoperative sepsis (1). With recent advances in therapy in the medical and surgical fields, the postoperative outcome has improved. Despite this progress, certain patients are still at a high risk of infection, followed by morbidity and mortality. Early markers of septic complications would be useful for the diagnosis and therapeutic management of patients with postoperative sepsis.

Cytokines are not only mediators of inflammation, but also play an important role in the regulation of the immune system. Reliable measurements of endogenous mediators, such as tumor necrosis factor-α (TNF-α), interleukin-8 (IL-8) and interleukin-10 (IL-10), have enabled us to clarify the pathway of this inflammatory response, and thus are important in the clinical setting (2).

TNF-α, a proinflammatory cytokine, is produced by macrophages and monocytes. It is a potent activator of neutrophils and endothelial cells, acting through two distinct TNF receptors. IL-8, a proinflammatory cytokine released by cells, including endothelial cells, monocytes and T cells, has been recognized as a relevant activator of leukocytes, and is related to adult respiratory distress syndrome (3). IL-10 is a potent antiinflammatory cytokine that reduces neutrophil adhesion to activated endothelial cells.

Acupuncture is a Chinese medicine treatment that involves inserting needles into specific sites, known as acupoints, on the body’s surface. The acupoints may be stimulated by different methods, such as manual needling, the delivery of electrical current or heat to the acupuncture needle, or by applying pressure or laser-generated heat to the acupoint. Attenuation of the inflammatory response reduces injury-induced immunosuppression and is relevant to functional recovery (4).

The aim of our study was to observe the effect of eletroacupuncture (EA) on cytokines and immune function and to investigate whether point specificity was present in EA.

## Materials and methods

### Patient management

This study adopted a randomized, controlled trial procedure, and 29 patients undergoing supratentorial craniotomy were enrolled. The study protocol was approved by the Institutional Review Board of Beijing Tiantan Hospital and written informed consent was obtained from all patients. The patients were aged between 18–60 years old, and the surgery had been assessed to be grade 1–2 by the American Society of Anesthesiologists (ASA). The following cases were excluded from this study: patients with immune, renal or central nervous system dysfunction, patients with congestive heart failure, exogenous hormone therapy (including steroids), prior experience of acupuncture, pregnancy, malnutrition, diabetes, malignancy, infection or inflammation.

No analgesics or tranquilizers were administered prior to surgery. Once in surgery, the peripheral intravenous infusion was initiated, and noninvasive blood pressure (NIBP), heart rate (HR), oxygen pulse saturation (SpO_2_) and bispectral index (BIS) were monitored. In group A, EA was applied to Hegu (LI4), Waiguan (TE5), Jinme (BL63), Taichong (LR3), Zusanli (ST36), Qiuxu (GB40), Tianzhu (BL10), Fengchi (GB20), Cuanzhu (BL2) and Yuyao (EX-HN4) on the side with the craniotomy. The needles were inserted to a depth of 0.75–1.5 cm at these acupoints. The electroacupuncture stimulation (EAS) was delivered via a HANS acupoint nerve stimulator (LH202H, Huawei Co., Ltd., Beijing, China), with a disperse-dense wave, 2 Hz/100 Hz in frequency, alternated once every 3 sec. The complete symmetric biphasic pulse was adopted. The stimulation intensity was in accordance with the maximal tolerance of patients and the EAS lasted from the induction of anesthesia until the end of surgery.

Group C was a general anesthesia control group. In this group, EA was not applied to patients, and other conditions were the same as those of the other two groups.

Group S was a sham acupoints group. In this group, EA was applied at 9 and 12 Cun above Kunlun (BL60), 7 and 10 Cun above Taixi (KI3) and 7 and 9 Cun above Shenmen (HT7) on the side with the craniotomy. The needles were inserted to a depth of 0.75–1.5 cm. EAS was delivered via a HANS acupoint nerve stimulator (LH202H, Huawei Co., Ltd.), using a disperse-dense wave, 2 Hz/100 Hz in frequency, alternated once every 3 sec. The complete symmetric biphasic pulse was adopted. The stimulation intensity was in accordance with the maximal tolerance of patients and EAS lasted from the induction of anesthesia until the end of surgery.

### Target-controlled infusion (TCI) of propofol and sufentanil

The induction plasma concentration of propofol was 5 μg/ml, and that of sufentanil was 0.5 ng/ml. While the patient was unconscious, the plasma concentration of propofol was reduced to 3.2 μg/ml and that of sufentanil to 0.3 ng/ml, and vecuronium bromide 0.1 mg/kg was administered at the same time. After muscle relaxation, tracheal intubation was performed. Mechanical ventilation with pure oxygen was applied, 10 ml/kg in tidal volume, 12 times/min in respiratory frequency and 1 l/min in oxygen flow. Vecuronium bromide (0.05 mg/kg) was intermittently administered performed to maintain muscle relaxation. Sufentanil concentration was adjusted to maintain the mean arterial pressure (MAP) and HR in the basic range of +10% to −20%. In cases of hypotension (MAP <20% of baseline), bradycardia (HR <50 beats/min) or hypertension (MAP >10% of baseline values), 6 mg ephedrine, 0.5 mg atropine or 0.2–0.5 mg nicardipine was administered, respectively.

### Sampling

Blood samples were obtained at the following time points: before anesthesia (T0), 4 h after induction of anesthesia (T1), 1 day after surgery (T2) and 2 days after surgery (T3) in SST™II advance tubes (Becton Dickinson, Oxford, UK) for cytokine and immunoglobin concentration testing.

### Multiplex cytometric bead assay

A cytometric bead assay kit (Becton Dickinson) was employed to measure levels of TNF-α, IL-8, IL-10, IgM, IgA, and IgG in plasma according to the manufacturer’s instructions.

### Statistical analysis

The department of epidemiology and hygienic statistics of Capital Medical University was responsible for data input and statistical analysis. SPSS 13.0 statistical software was adopted. Results are expressed as the mean ± SD. Data were analyzed using repeated-measures analysis of variance and separate effect analysis. In the analysis, Mauchly’s test was used to judge whether there were correlations between the repeatedly measured data. When P<0.05, we used Greenhouse-Geisser to correct the results. Differences were considered to be statistically significant at P<0.05. There were significant differences between group C and group A or group C and group S for the baseline value of IgA and IgM, so we used the D-value to T0 to compare the differences among the three groups at the other time points.

## Results

### General condition

The randomized controlled trial had 29 participants, and no subject withdrew from the trial. Patient characteristics and surgical data are provided in Table I. There were no differences in the arterial blood pressure or HR among the groups prior to, during or after EAS. There was no surgical morbidity or mortality.

### Comparison within the groups

IL-10 levels in peripheral blood increased significantly compared with those at T0 at 1 and 2 days after surgery in groups A and S, and 4 h after the induction of anesthesia and 1 day after surgery in group C. The IL-8 levels in peripheral blood increased significantly compared with those at T0 at 1 and 2 days after surgery in groups A and S. The IgA levels in peripheral blood decreased significantly compared with those at T0 at 4 h after the induction of anesthesia, and 1 and 2 days after surgery in group C (Table II).

### Comparisons among the three groups

**Figure 1:**
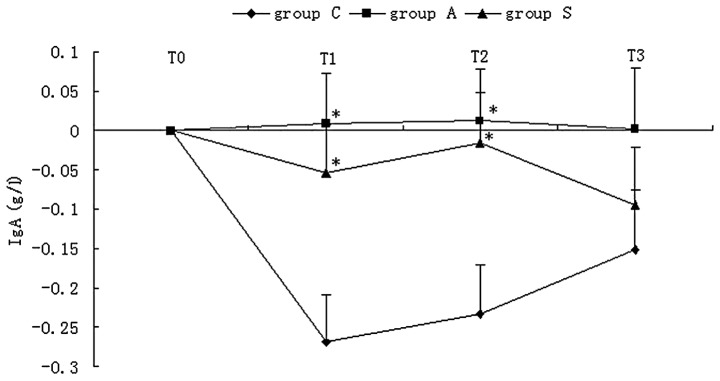
Variations in blood IgA level after anesthesia were compared among the three groups. ^*^Compared with group C, there was a significant difference between group C and group A or group C and group S (P<0.05).

When comparing the levels of serum cytokines during the craniotomy, we discovered that the peripheral blood IL-8 levels in groups A and S increased significantly compared with that of group C at 1 and 2 days after surgery (Table II). When comparing the levels of serum immunoglobulin during the craniotomy, we observed that the peripheral blood IgA levels in group C had decreased significantly compared with those of groups A and S at 4 h after induction of anesthesia and 1 day after surgery (Fig. 1).

## Discussion

Surgery and the resultant stress response lead to a suppression of immune function (5). The results of our study indicated that EA improves the immune function suppressed by surgery. Our study found that the peripheral blood IgA of patients in group C decreased significantly compared with those of groups A and S 4 h after the induction of anesthesia and 1 day after surgery. We also noted that the IL-10 and IL-8 levels in peripheral blood were increased significantly compared with those at T0 at 1 day and 2 days after surgery in groups A and S.

IgG may have a significant protective function (6). IgG is important in bacterial and viral defense; therefore, it may be advantageous in preventing postoperative infection. A previous study reported that IgG was modulated in obese women treated with EA compared with women treated with placebo EA and restricted diet only (7); however, there was no observed difference amongst the three groups in our study.

Another study investigated the application of EA in 70 volunteers, and observed changes in immunoglobulin levels in the serum, saliva and gingival sulcus fluid (8). The results of the study showed that 30 min and 24 h after acupuncture treatment, saliva IgA levels were significantly increased in volunteers whose IgA levels had previously been low. When acupuncture was applied daily for 2 weeks, the study observed that saliva IgA levels were increased significantly by ~20% compared with the baseline value. This is similar to the results from our own study.

Kho *et al* observed 29 male patients during and 6 days after upper abdominal surgery, and changes in IgA, IgM and IgG levels in the peripheral blood were recorded. The surgery was performed under two different anesthetic techniques (9): group 1 received acupuncture and small doses of fentanyl and group 2 received moderate doses of fentanyl. After surgery, the level of immunoglobulin decreased in the two groups. IgA and IgG recovered by the sixth day after surgery in the two groups, and IgM recovered by the fourth day. Acupuncture and transcutaneous stimulation analgesia in patients undergoing major abdominal surgery did not affect the immune system, which was measured by the concentrations of immunoglobulin either during or after surgery. In our study, we observed that peripheral blood IgA of the patients in group C had decreased significantly compared with those of groups A and S 4 h after the induction of anesthesia and 1 day after surgery, which showed that EA in patients undergoing supratentorial craniotomy may affect the immune system as measured by the concentrations of immunoglobulins.

In a previous study, rats received EA at the Zusanli (ST36) and Neiguan (PC6) acupoints, or electrical stimulation at sham points, for 30 min prior to stimulation with either 5 mg/kg LPS intravenously or normal saline (10). Plasma cytokines were assessed 240 min after either LPS or normal saline injection. The results demonstrated that EA pretreatment significantly decreased LPS-induced plasma TNF-α and IL-1β levels and increased the plasma IL-10 level. The findings suggested that EA pretreatment at the ST36 and PC6 acupoints attenuated the LPS-induced inflammatory response. However, our study demonstrated that the IL-8 levels in groups A and S were significantly increased when compared with that of group C at 1 and 2 days after surgery. This showed that EA is also an injury to the body as well as an advantage.

Numerous acupuncture studies use a sham acupoints group as a control group, but it has been found that sham acupoints have similar effects to those of true acupoints. Furthermore, the results of pain-related studies have not supported the concept of point specificity (11). However, certain studies evaluating the response to stimulation of multiple points on the body surface have shown that point-specific actions are present. Therefore, the existence of point specificity in acupuncture remains controversial. In our study, we observed that there was no difference between groups A and S for IL-8 and IgA levels, which confirms that point specificity in EA was not present.

Our study indicated that EA, in addition to its analgesic effects, prevented the decrease of immunoglobulin after surgery. The study also suggested that the stimulation of non-acupoints elicits effects similar to those of stimulation of true acupoints. Further studies in this area are required in order to detect the mechanisms. Acupuncture-drug combined anesthesia should be used to partially improve immune suppression after surgery.

## Figures and Tables

**Table I:** Patient characteristics and surgical data.

	Group C	Group A	Group S
Gender (male/female)	5/5	5/4	4/6
Age (years)	42±10	43±9	39±7
Weight (kg)	67±10	65±9	68±11
Height (cm)	165±11	67±23	163±17
Duration of surgery (min)	253±43	241±49	233±51
ASA class (I/II)	8/2	7/2	7/3
Tumor type (glioma/meningioma/other tumors)	4/4/2	4/3/2	4/4/2

ASA, American Society of Anesthesiologists.

**Table II:** Levels of cytokines and immunoglobulins following surgery.

	Group	Before anesthesia	4 h after induction of anesthesia	1 day after surgery	2 days after surgery
TNF (pg/ml)	Group C	1.51±0.41	1.71±0.16	1.61±0.22	1.4±0.38
	Group A	2.53±0.43	2.82±0.16	3.09±0.24	3.82±0.4
	Group S	3.44±0.41	2.75±0.16	3.15±0.22	2.99±0.38
IL-10 (pg/ml)	Group C	1.28±0.21	4.83±1.32^a^	3.97±1.04^a^	1.66±0.53
	Group A	2.53±0.22	3.16±1.4	7.18±1.1^a^	5.69±0.56^a^
	Group S	2.82±0.21	3.04±1.32	6.36±1.04^a^	4.01±0.53^a^
IL-8 (pg/ml)	Group C	11.45±5.67	10.9±1.67	17.48±46.61	12.36±60.33
	Group A	8.96±6.52	16.73±1.76	111.52±49.14^a^^b^	228.61±63.59^a^^b^
	Group S	38.08±15.67	13.54±1.67	119.18±46.61^a^^b^	174.28±60.33^a^^b^
IgM (g/l)	Group C	1.47±0.11	1.16±0.1	1.29±0.13	1.38±0.12
	Group A	0.19±0.11	0.19±0.11	0.19±0.14	0.18±0.13
	Group S	0.32±0.11	0.26±0.1	0.3±0.13	0.26±0.12
IgA (g/l)	Group C	4.16±0.41	2.77±0.37^a^	2.89±0.35^a^	3.25±0.31^a^
	Group A	0.61±0.43	0.64±0.39^b^	0.65±0.37^b^	0.63±0.32
	Group S	1.05±0.41	0.88±0.37^b^	0.98±0.35^b^	0.71±0.31
IgG (g/l)	Group C	0.53±0.08	0.47±0.07	0.44±0.08	0.45±0.07
	Group A	0.42±0.08	0.42±0.08	0.42±0.09	0.41±0.08
	Group S	0.46±0.08	0.46±0.07	0.46±0.08	0.46±0.07

Values are expressed as the mean values ± SD.

a Compared with before anesthesia, there was a significant difference within the group, (P<0.05);

b compared with group C, there was significant difference between group C and group A or group C and group S (P<0.05).

TNF, tumor necrosis factor; IL, interleukin.
